# Supplementary material for: Conditional chemoconnectomics (cCCTomics) as a strategy for efficient and conditional targeting of chemical transmission
Source: eLife. 2024 Apr 30;12:RP91927. doi: 10.7554/eLife.91927 (PMC11060718; doi:10.7554/eLife.91927)
Supplement: Figure 3—source data 3. [file elife-91927-fig3-data3.zip › Figure 3 source data3 part 2.zip!/Figure 3-Source data 3-part2/1053f2-1-CRISPResso_Report_y3hlioj1.zip!/CRISPResso_on_1053f2_4/9.Alleles_frequency_table_around_sgRNA_tggtcctgaccatctacatt.pdf]

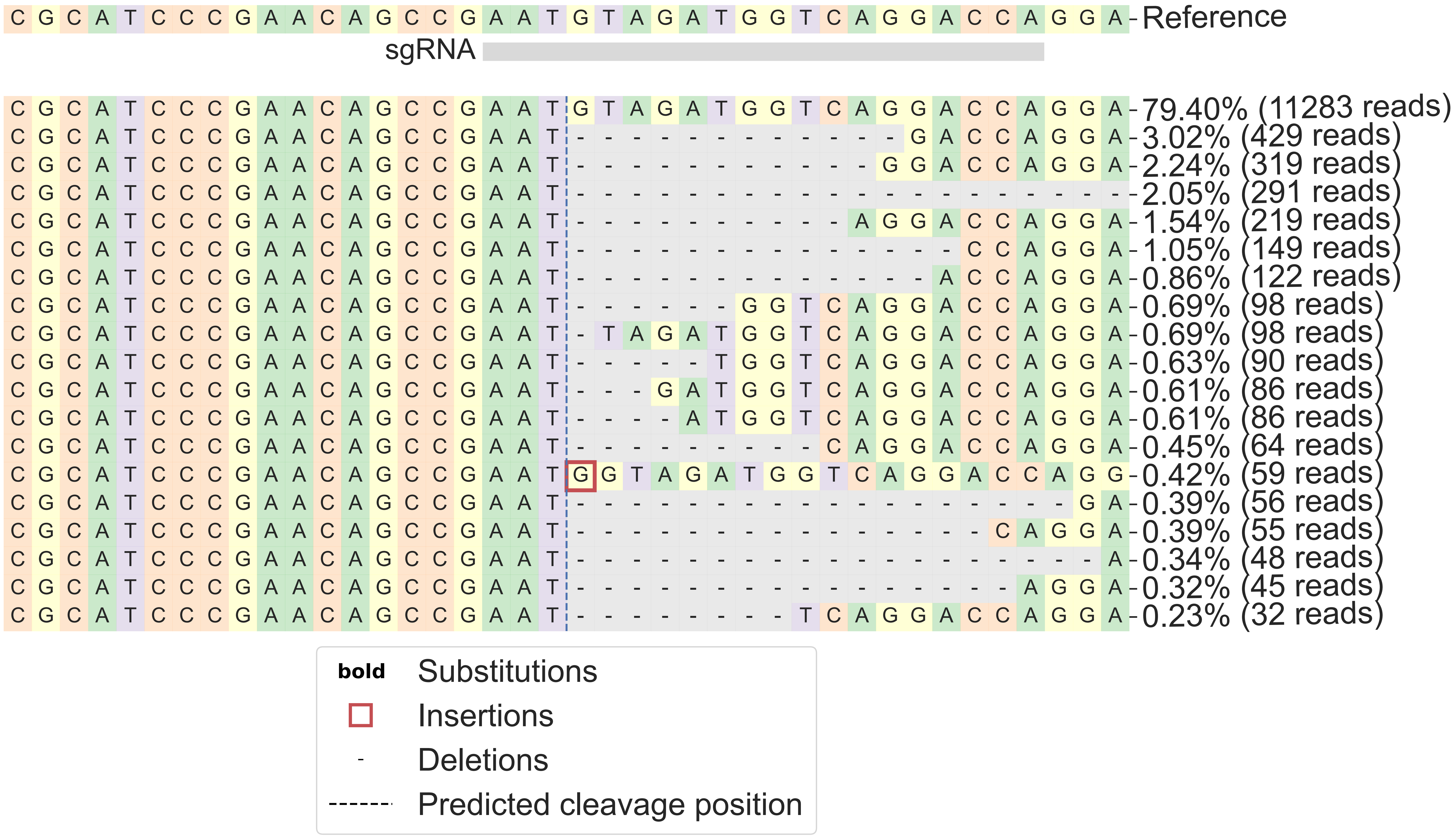
CGCATCCCGAACAGCCGAATGTAGATGGTCAGGACCAGGA Reference
sgRNA
CGCATCCCGAACAGCCGAATGTAGATGGTCAGGACCAGGA 79.40% (11283 reads)
CGCATCCCGAACAGCCGAAT------------GACCAGGA 3.02% (429 reads)
CGCATCCCGAACAGCCGAAT-----------GGACCAGGA 2.24% (319 reads)
CGCATCCCGAACAGCCGAAT-------------------- 2.05% (291 reads)
CGCATCCCGAACAGCCGAAT----------AGGACCAGGA 1.54% (219 reads)
CGCATCCCGAACAGCCGAAT--------------CCAGGA 1.05% (149 reads)
CGCATCCCGAACAGCCGAAT-------------ACCAGGA 0.86% (122 reads)
CGCATCCCGAACAGCCGAAT------GGTCAGGACCAGGA 0.69% (98 reads)
CGCATCCCGAACAGCCGAAT-TAGATGGTCAGGACCAGGA 0.69% (98 reads)
CGCATCCCGAACAGCCGAAT-----TGGTCAGGACCAGGA 0.63% (90 reads)
CGCATCCCGAACAGCCGAAT---GATGGTCAGGACCAGGA 0.61% (86 reads)
CGCATCCCGAACAGCCGAAT----ATGGTCAGGACCAGGA 0.61% (86 reads)
CGCATCCCGAACAGCCGAAT---------CAGGACCAGGA 0.45% (64 reads)
CGCATCCCGAACAGCCGAATGGTAGATGGTCAGGACCAGG 0.42% (59 reads)
CGCATCCCGAACAGCCGAAT------------------GA 0.39% (56 reads)
CGCATCCCGAACAGCCGAAT---------------CAGGA 0.39% (55 reads)
CGCATCCCGAACAGCCGAAT-------------------A 0.34% (48 reads)
CGCATCCCGAACAGCCGAAT----------------AGGA 0.32% (45 reads)
CGCATCCCGAACAGCCGAAT--------TCAGGACCAGGA 0.23% (32 reads)
bold Substitutions
Insertions
- Deletions
------ Predicted cleavage position
